# Peripheral Nervous System Reconstruction Reroutes Cortical Motor Output—Brain Reorganization Uncovered by Effective Connectivity

**DOI:** 10.3389/fneur.2018.01116

**Published:** 2018-12-18

**Authors:** Ahmad Amini, Florian Ph.S. Fischmeister, Eva Matt, Robert Schmidhammer, Frank Rattay, Roland Beisteiner

**Affiliations:** ^1^Study Group Clinical fMRI, Department of Neurology, Medical University of Vienna, Vienna, Austria; ^2^Highfield MR Centre, Medical University of Vienna, Vienna, Austria; ^3^TU-BioMed Association for Biomedical Engineering, Vienna University of Technology, Vienna, Austria; ^4^Ludwig Boltzmann Institute for Experimental and Clinical Traumatology, Vienna, Austria; ^5^Institute of Psychology, University of Graz, Graz, Austria

**Keywords:** Dynamic Causal Modeling (DCM), functional magnetic resonance imaging (fMRI), phrenic nerve, brachial plexus avulsion, peripheral nerve reconstruction

## Abstract

Cortical reorganization in response to peripheral nervous system damage is only poorly understood. In patients with complete brachial plexus avulsion and subsequent reconnection of the end of the musculocutaneous nerve to the side of a phrenic nerve, reorganization leads to a doubled arm representation in the primary motor cortex. Despite, homuncular organization being one of the most fundamental principles of the human brain, movements of the affected arm now activate 2 loci: the completely denervated arm representation and the diaphragm representation. Here, we investigate the details behind this peripherally triggered reorganization, which happens in healthy brains. fMRI effective connectivity changes within the motor network were compared between a group of patients and age matched healthy controls at 7 Tesla (6 patients and 12 healthy controls). Results show the establishment of a driving input of the denervated arm area to the diaphragm area which is now responsible for arm movements. The findings extend current knowledge about neuroplasticity in primary motor cortex: a denervated motor area may drive an auxilliary area to reroute its motor output.

## Introduction

A disturbance of arm functions can occur as a result of various lesions within the central and/or peripheral nervous system. To advance therapeutic progress, a comprehensive understanding of the neuroplastic mechanisms which allow restoration of upper limb function after such a disturbance is essential. There is already a large body of functional neuroimaging literature on the flexibility of cortical reorganization in response to central nervous system damage. Typical mechanisms include an overactivation of secondary brain areas or of homologous (contralateral to the damaged side) brain areas, and a changed pattern of driving/inhibiting interactions within the somatosensory network [for review (1)] or the motor network [for review (2)]. Such cortical reorganization even occurs in the case of extreme central nervous system damage such as hemispherectomy (3). In contrast, only few neuroimaging studies exist on cortical reorganization in response to *peripheral* nervous system damage. With peripheral damage brain neuroplasticity is not induced by brain lesions—instead, the changed information flow between the somatic periphery and the brain constitutes the driving factor. It is important to note that neuroplasticity in response to peripheral nerve injury happens in a completely healthy brain. Neuroplastic changes are mostly found in non-primary brain areas but can also involve functional changes of primary somatosensory and primary motor areas contra- and ipsilateral to the injured limb (4–7). This is interesting since primary motor areas are highly specialized cortices, with dedicated somatotopy; for instance, even movements of single fingers are functionally separable by fMRI (8). Furthermore, the primary motor cortex is an essential cortex—its function is not recoverable after destruction.

In previous literature, 4 different types of cortical reorganization after peripheral damage of the upper limb have been reported. Taking the perspective of the primary motor cortex they may be described as follows: (1) The task for the locally specialized primary motor cortex does not change. The goal of reorganization is to achieve the original upper limb function [e.g., after reconnection of a transected median nerve (9, 10)]. (2) The task for the locally specialized primary motor cortex does not change. However, the final output has to be adapted to a new effector. The goal of reorganization is to achieve a similar upper limb function but with a changed effector [e.g., after heterotopic hand replantation (11)]. (3) The task for the locally specialized primary motor cortex does change. For example, instead of generating arm extension, arm flexion now has to be performed. The goal of reorganization is to replace a specific motor function by a new motor function [e.g., after connecting a C7 root to the musculocutaneus nerve (12), thereby sacrificing the original C7 function]. (4) The task for the locally specialized primary motor cortex is lost. The goal of reorganization is to adapt to the lost upper limb function [e.g., after amputation (13)].

In addition to these four well-known types of cortical reorganization after peripheral nervous system damage, an initial piece of evidence has recently been published that neuroplasticity in primary motor cortex may go even beyond that. This evidence has been described in a therapeutically difficult group of patients—patients with brachial plexus avulsion. Here, various concepts for nerve transfer exist to allow at least partial regain of arm function. When connecting the ending of a denervated musculocutaneous nerve to the side of an intact phrenic nerve, the task for the locally specialized primary motor cortex does not change. However, a new task has to be added. Now, the cortical diaphragm representation has to perpetuate control of breathing but add independent control of arm flexion (14, 15). Initially, the biceps muscle is stimulated and forced inspiration procedures are used to start innervation of the muscle (“breathing muscle”). Later, independent innervation of diaphragm and arm flexion is intensively trained. Typically, it takes 12–18 months after surgery, to achieve independent breathing and arm flexion patterns. Currently it is not known how this functional reorganization of the motor network is realized. A promising tool for investigating clinical network changes is effective connectivity fMRI (16–24). In this study, we provide a first Dynamic Causal Modeling (DCM) examination of the motor network changes accompanying such a peripheral reconstruction phenomenon. The goal is to investigate which mechanisms underlie the reestablishment of arm function via a doubled arm representation in the primary motor cortex.

## Materials and Methods

### Participants

Six right-handed patients (5 males and 1 female, age range: 26–47) with end-to-side coaptation of a traumatic right or left brachial plexus avulsion (3 with left arm injuries and 3 with right arm injuries; mean time period post-surgery 1 year) (see Table 1) participated in this study. Although a final regeneration state probably has not yet been achieved with every patient, all patients could generate a voluntary contraction of the diseased biceps muscle, which was independent from breathing (as documented by EMG recordings, cf. 14). Twelve right-handed healthy subjects matched for age and gender served as control group (see Table 2). None of the subjects had any history of neurological or psychiatric illness except for the brachial plexus lesion in the patients group. Written consent and ethical approval was received from all controls and patient subjects before they entered the study.

**Table 1:** Detailed characterization of the phrenic nerve patients.

**Patients**	**Age_Range**	**Injured arm**	**Accident date**	**Operation date**	**Scanning date**
1	25–30	Left arm	June 2010	February 2012	February 2013
2	30–35	Right arm	August 2011	February 2012	March 2013
3	35–40	Left arm	May 2009	April 2011	February 2012
4	35–40	Right arm	June 2010	December 2010	March 2014
5	35–40	Left arm	September 2010	February 2011	May 2013
6	40–47	Right arm	March 2012	February 2013	December 2013
Age_Average				36.8	
Age_Median				37	
Age_Interquartile range (IQR)				3.5	

**Table 2:** Detailed characterization of the controls.

**Control**	**Age_Range**	**Scanning date**
1	25–30	December 2013
2	25–30	September 2015
3	25–30	December 2013
4	30–35	August 2015
5	30–35	July 2014
6	30–35	September 2015
7	35–40	July 2014
8	35–40	August 2015
9	35–40	January 2014
10	40–47	September 2015
11	40–47	January 2014
12	40–47	July 2014
Age_Average		34.6
Age_Median		34.5
Age_Interquartile range (IQR)		8.25

### Experimental Design

The fMRI study involved three different tasks: (1) isolated elbow flexion of the patients' diseased arm and healthy subjects right arm. For three patients the diseased arm was the right arm. (2) isolated elbow flexion of the other arm and (3) forced abdominal inspiration with keeping both arms relaxed. The amplitude of the elbow flexion was minimized by the instruction “lift the forearm only slightly but generate a strong muscle tension without change of breathing.” Before the measurement, elbow flexion was trained and successful performance was documented outside of the scanner.

Each task was performed separately in a blocked fashion consisting of 3 ON and 4 OFF phases of 20 s duration per trial. Patients performed 20, controls 24 successful fMRI trials. During ON phases repetitive movements (lift-relax or inspiration-expiration) were triggered by a visual command with 4 arm liftings/inspirations per ON phase. Subjects were continuously monitored by an operator from within the scanner room during each acquisition to ensure accurate performance.

### Data Acquisition

The subjects were measured on a 7T Siemens MRI system (Siemens, Erlangen, Germany) with a 32-channel phased array head coil. A single-shot gradient-echo EPI pulse sequence was used to acquire functional images (FOV of 230, TE/TR of 22/2500 ms, matrix size of 128 × 128 and 39 interleaved axial slices (1.8 mm thickness, 3 mm gap) aligned to the AC-PC line). In addition, high resolution T1-weighted anatomical (inversion-recovery) images were acquired: FOV of 230, TE/TR of 3.34/4460 ms, matrix size of 307 × 320 and voxel dimensions of 0.7 × 0.7 × 0.7 mm. The head of each participant was fixed by an individually prepared plaster cast helmet to reduce head movement artifacts (25). For each patient, 20 successful fMRI trials of 140 s duration (3 ON, 4 OFF phases, 56 volumes per trial) were acquired—all within a single experimental session (total duration about 1.5 h). Trials with insufficient compliance or large body movements were discarded and repeated. Per experimental session, the following trials were performed: 8 trials forced abdominal inspiration, 8 trials elbow flexion injured arm and 4 trials elbow flexion healthy arm. Controls performed 24 fMRI trials: 12 forced abdominal inspiration, 6 elbow flexion of the right arm, 6 elbow flexion of the left arm. To allow a group comparison of healthy and diseased arm motor networks, the images of the right-injured arm patients were flipped.

### Image Preprocessing

The fMRI image analysis was carried out using SPM12 (Wellcome Trust Center for Neuroimaging, Institute of Neurology, UCL, UK, http://www.fil.ion.ucl.ac.uk/spm). Preprocessing included slice-time correction (26), realignment (accepting only sessions with a translation of < 2.5 mm in all three directions and with rotation < 1° around all three axes) and smoothing using a 5 mm full-width-at-half-maximum Gaussian filter. The anatomical images were co-registered to the mean EPI and then SPM12 segmentation was applied to derive the individual tissue classes.

Preprocessed data (20 trials for patients and 24 trials for controls) were then concatenated into one single run to form a single time series for each individual.

### Statistical Analyses

The first three dimensions (A3, eigenvariates) of the signals within the main tissue classes (white matter, cerebrospinal fluid, skull/bones, and soft tissue) were extracted by using the a CompCor (27) implementation in the REX toolbox (http://web.mit.edu/swg/software.htm).

Single subject brain activation was modeled using a general linear model (GLM) (28) with task specific regressors representing the ON phases of this specific task, convolved with the hemodynamic response function. Twenty nuisance regressors were added to each trial: six movement correction parameters, 12 aCompCor regressors, one block regressor for modeling each trial mean and one transition regressor (29) for modeling the transition between the trials (total number of regressors = no. of sessions × 20). The data were high pass filtered with a frequency cutoff period at 128 s to remove low frequency drifts.

**Figure 1:**
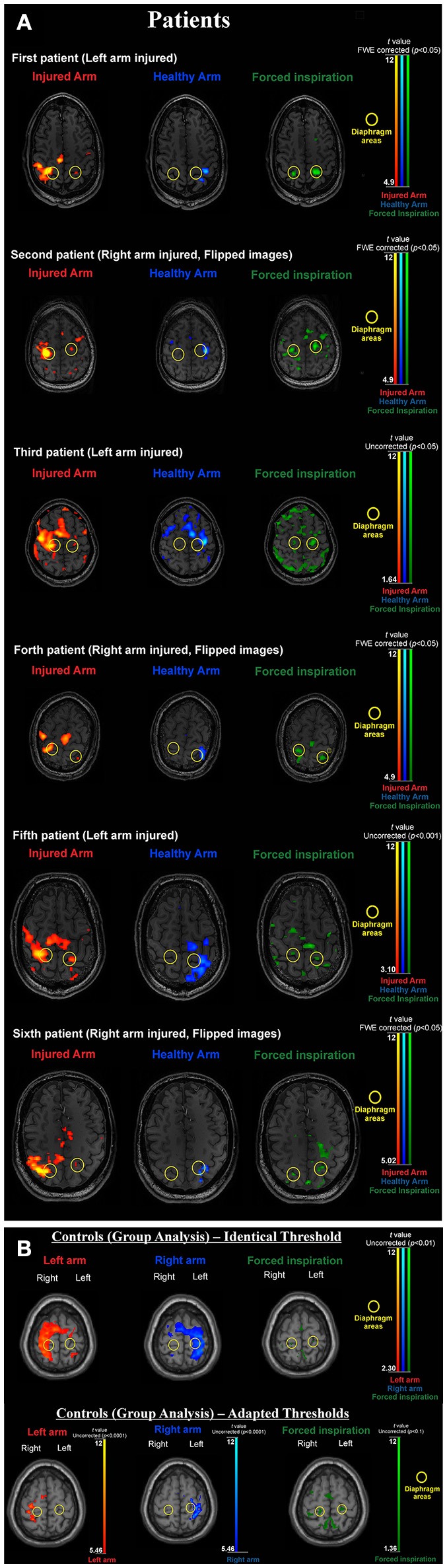
**(A)** BOLD activation results for all individual patients. During forced inspiration artifact levels are increased. Thresholds are adapted according to the individual contrast to noise situation (which largely varied in this difficult patient group). Note that with activation of the injured arm the *diaphragm area* of the non-dominant hemisphere is always activated and the *arm area* activation of the dominant hemisphere always extends to the *diaphragm area* of the dominant hemisphere (yellow circles). Both features are absent when activating the healthy arm. **(B)** BOLD activation results for the healthy control group. Upper part: due to low contrast to noise ratio with the artifact prone inspiration task left and right arm clusters overlap diaphragm clusters when using the same threshold. Lower part: adapted thresholds [compare (36)] reveal, that arm activations are generated in arm areas (inverted omega structure) and do not include the diaphragm representations (yellow circles) and diaphragm activations do not include arm areas. For the group image, brains were normalized and the 12 tissue regressors (aCompCor) were not added for first level modeling.

As peak activation varied across subjects we followed the approach of Klinge et al. (29) and Li et al. (30). First critical motor network areas were defined purely on functional neuroanatomical criteria: i.e., the phrenic nerve (diaphragm) representation is located medial to the hand representation (inverted omega) in the primary sensorimotor cortex (see Figures 1A,B). Task based fMRI clusters and their peak activations within the primary motor cortex were individually identified. The regions of interest (ROI) comprised the left and right supplementary motor areas (SmaL and SmaR), left and right arm primary motor cortex areas (ArmL and ArmR), and left and right diaphragm primary motor cortex areas (DiaL and DiaR).

Activations within these ROIs served as a basis for the detection of individual peak *t*-value voxels resulting in 6 peak voxels, one for each ROI. Then the time courses of these ROIs were extracted separately for every participant using SPM12's volume of interest (VOI) batch script (VOI radius = 8 mm, single-subject significant threshold *p* < 0.05 FWE, first eigenvariate used as summary statistic).

### DCM Analysis

Although pure BOLD fMRI data provide a clear picture about brain activations they do not allow inferences which activations are correlated or which activations may drive others. The latter is possible with Dynamic causal modeling (DCM). DCM12 (31) as implemented in SPM12 allows inferences about the architecture of distributed networks in the brain in terms of effective connectivity. Commonly, a bilinear approximation is applied in DCM, because this reduces the number of parameters to three inputs: (1) direct/extrinsic input, which has an influence on the states, (2) intrinsic/latent connections, which couples one area to the state of others, and (3) modulatory input, which modulates the intrinsic coupling.

#### Model Space Construction

##### Intrinsic connections

**Figure 2:**
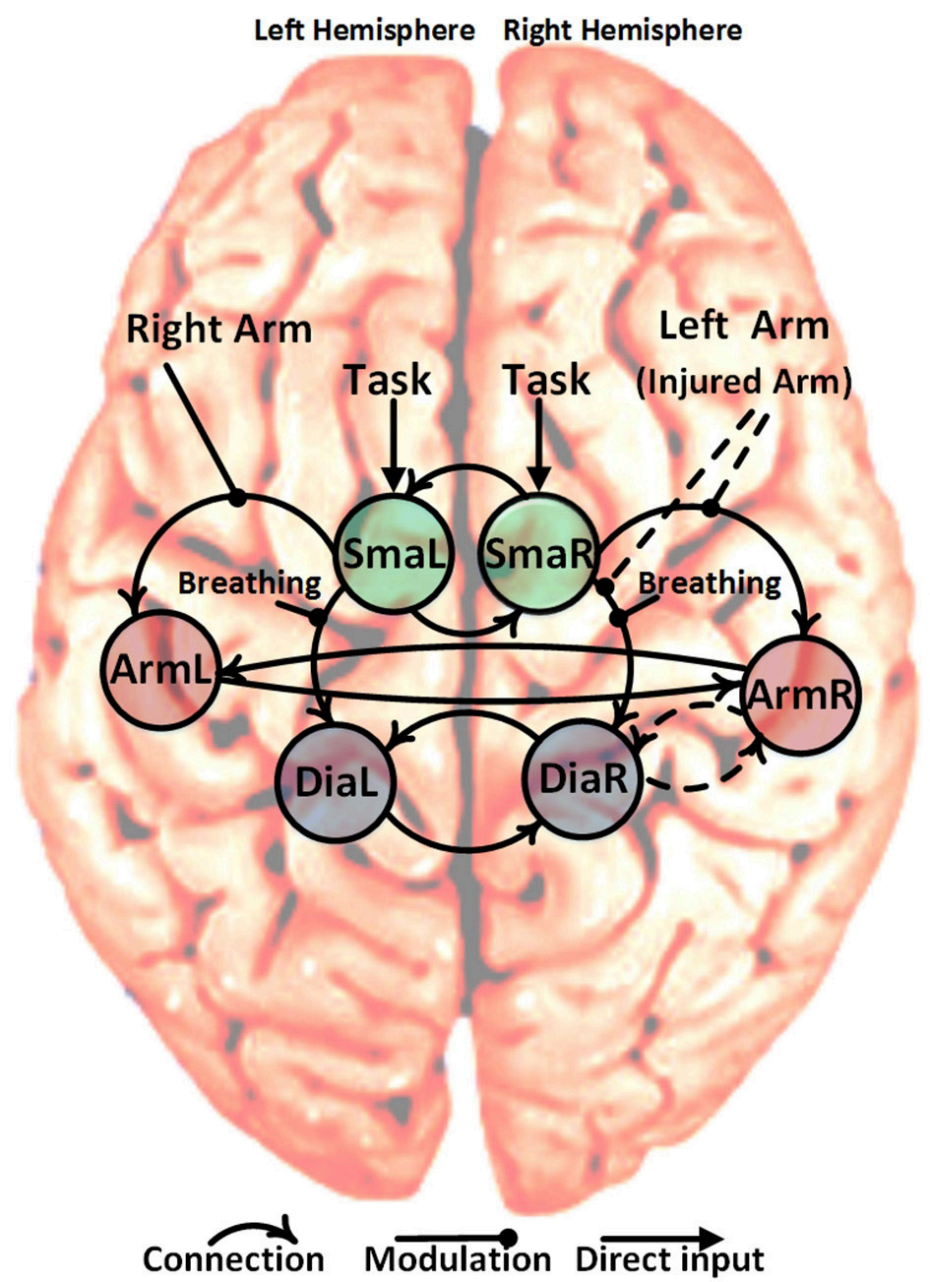
Schematic representation of the six specified ROIs, solid curved arrows show the fixed connection and dashed curved arrows show variable connections (2^2^ = 4 different models). The non-curved solid arrows and solid lines show the fixed direct and modulatory inputs, respectively. The non-curved dashed lines show the variable modulatory input of the left arm (injured arm) with three possibilities: input to connection (SmaR to ArmR), or input to connection (SmaR to DiaR) and or both of them simultaneously. This gives a total of 12 different candidate dynamic causal models (4 connections ^*^ 3 left arm modulatory inputs).

Within our patient population, the arm area of the primary motor cortex corresponding to the injured upper limb is completely denervated. Despite this, the arm area is strongly activated during limb movement after phrenic nerve connection (14) but not during breathing (see Figure 1). Due to its denervation, the arm area no longer has a peripheral output. Our primary hypothesis is that patients' M1 cortices establish a new functional connection of upper limb M1 (primary motor cortex) with diaphragm M1 in the injured hemisphere (ArmR to DiaR). The new task of ArmR would be to activate DiaR for generation of arm movements. In our DCM model we realized this hypothesis with a feed-back control system between ArmR and DiaR allowing 4 different structural models (Figure 2, dashed arrows):

1. Forward connection ArmR-DiaR and backward connection DiaR-ArmR (feed-back control system)
2. Forward connection ArmR-DiaR only
3. Backward connection DiaR-ArmR only
4. No connection between ArmR and DiaR

Besides the assumption of this new intracortical connectivity (expected to be absent in our controls), our model also included all of the well-established physiological connections between primary and secondary motor areas, namely: SMA driving primary motor areas and interhemispheric connections between motor areas. These were established as fixed connections across all models (Figure 2, solid arrows).

##### Modulation of connections and direct input

Concerning modulations of the connections described above, three equivalent hypotheses are plausible for moving the injured arm:

1. SmaR increases activation of DiaR since DiaR has to fulfill 2 instead of 1 motor tasks
2. SmaR increases activation of ArmR which then activates DiaR for injured arm movements
3. Both effects occur

Combining the 4 possible intrinsic connections with the 3 possible modulations results in 12 models which were tested in the patient and control groups. This was done using four conditions in each case:

1. Task: this indicated the start of any of the experimental tasks (elbow flexion of the right arm, elbow flexion of the left arm and forced abdominal inspiration) and represented a direct input to the left and right supplementary motor areas (SmaR and SmaL). This was created by pooling and modeling all the experimental tasks.
2. Breathing (upon instruction to perform forced inspiration): this was used to modulate the strength of the forward connection from SmaL to DiaL and from SmaR to DiaR.
3. Right arm flexion: this was used to modulate the strength of the forward connection from SmaL to ArmL.
4. Left arm flexion (or injured arm for patients): this involved three possibilities, (i) modulate the strength of the forward connection from SmaR to ArmR, (ii) modulate the strength of the forward connection from SMAR to DiaR, or (iii) modulate both of those connections simultaneously (dashed arrows in Figure 2).

#### Model Comparisons and Bayesian Parameter Averaging

After estimation of the models a Bayesian model selection (BMS) approach was used for both the patient and the control group, to select the model with the greatest evidence within each group. BMS can proceed in a fixed effects (FFX) or random effects (RFX) method (32, 33). According to our assumption that the optimal model structure is a fixed effect in each group (34) and also the small number of subjects (35), the FFX method would have been preferable here, but for the sake of comparison with other studies, the RFX method was also employed.

For inference of the connectivity parameters, Bayesian Parameter Averaging (BPA) was used to compute the average parameter estimates (group-mean connection strengths and their probabilities) for each group's winning model. BPA computes a joint posterior density for the entire group by combining the individual posterior densities, treating the posterior from one subject as the prior for the next. There are four distinct advantages for using BPA. Firstly, it accounts for posterior covariance among the parameters. Secondly, computation is easy and efficient under Gaussian assumptions about the posteriors and thirdly, it produces a single posterior density for the entire group that can be used for Bayesian inference (33, 34) and finally this method is valid also for small numbers of subjects where classical statistical approaches are inappropriate (35).

## Results

### Significant Group Differences in Model Structure

**Figure 3:**
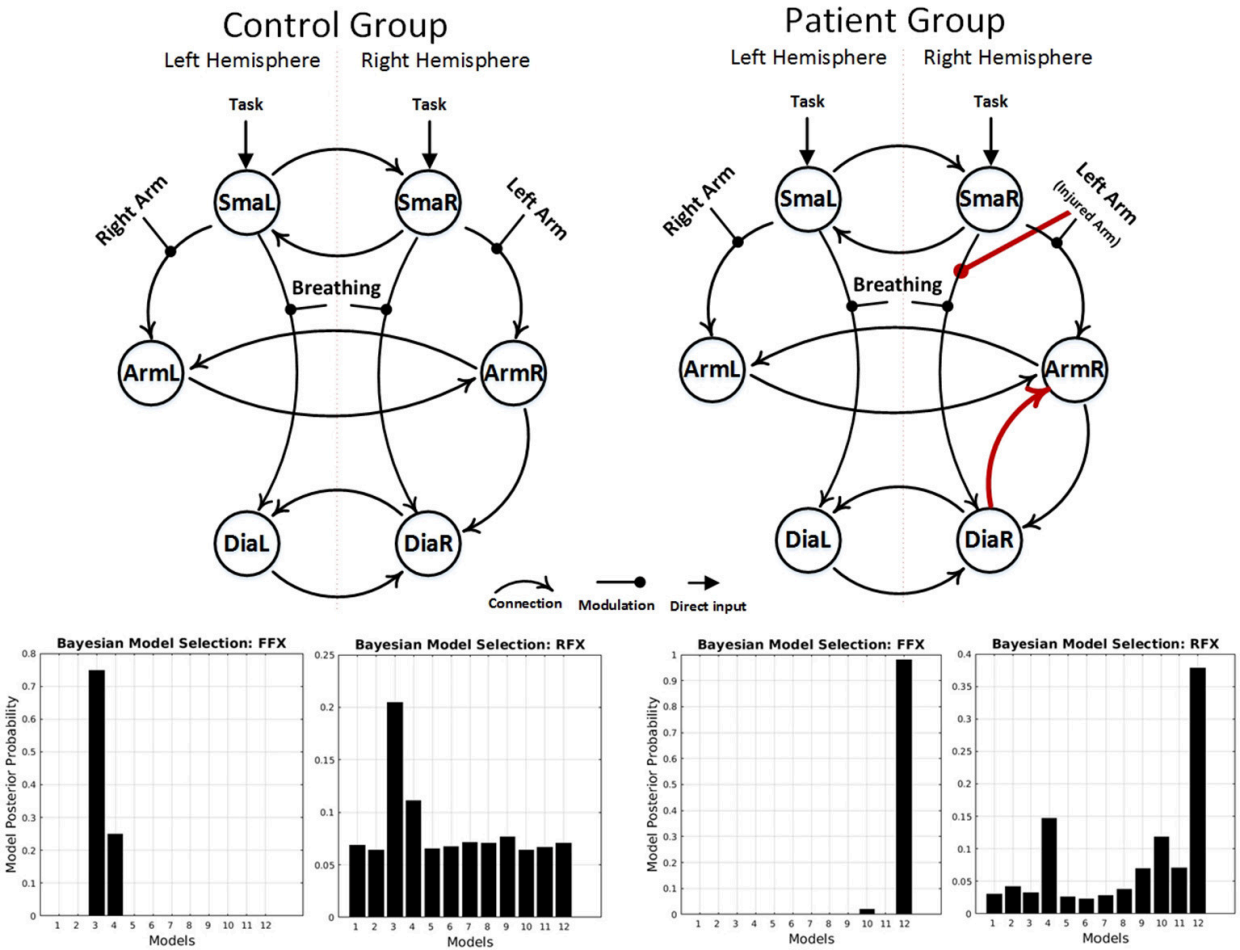
Results of the BMS analyses, under FFX and RFX assumptions, showing the winning models of the control group **(left)** and the patient group **(right)**. The result showed a group difference in model structure. Only the patient winning model shows an intracortical diaphragm to arm connection and left arm modulation to the forward connection from right SMA to right diaphragm area (red connections).

fMRI BOLD results are shown in Figures 1A,B. After applying the FFX and RFX, the results in each case show the same significant structural difference between the patient and control winning models (Figure 3 shows the controls and patients winning models and FFX and RFX model posterior probabilities). Only the patients winning model shows the following connections, which the control group did not show:

- *Forward connection from right diaphragm area (DiaR) to right primary motor cortex (ArmR)*
- *Left arm flexion (or injured arm for patients) modulation to the forward connection from right supplementary motor area (SmaR) to right diaphragm area (DiaR)*.

### Behavioral Group Differences in BPA

**Figure 4:**
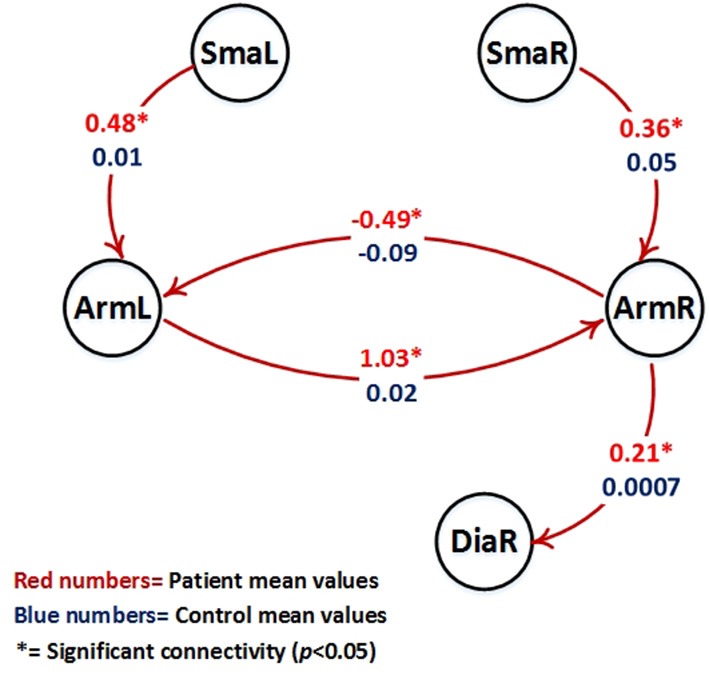
Visualization of parameter estimates from Table 3 (patients with red numbers). Only intrinsic connections which show a different group behavior are shown (all these connections are significant only in the patient group (*p* < 0.05, marked by ^*^) and are not significant in the control group). Note that patients right hemispheric arm area (ArmR) drives the right hemispheric diaphragm area (DiaR) to support movement of the diseased left arm.

Table 3 shows all parameters of the BPA analysis. To evaluate connections that differ between patients and controls, all connections that are significant (*P* < 0.05) only for one of the groups are marked by ^*^. Connections or modulatory inputs for which the groups show a different model structure are marked by ^**^ (compare Figures 3, 4).

**Table 3:** Patient and control group BPA results.

**Connections**	**Control**		**Patients**		**Different group behavior**
	**Mean**	***P*-value of BPA output**	**Mean**	***P*-value of BPA output**	
**INTRINSIC CONNECTIVITY (A)**					
SmaL to SmaR	0.55	1	0.75	1	
SmaL to ArmL	0.01	0.52	0.48	1	^*^
SmaL to DiaL	0.15	0.96	0.26	1	
SmaR to SmaL	−0.65	1	-0.76	1	
SmaR to ArmR	0.05	0.81	0.36	1	^*^
SmaR to DiaR	0.19	1	0.11	0.96	
ArmL to ArmR	0.02	0.55	1.03	1	^*^
ArmR to ArmL	−0.09	0.82	-0.49	1	^*^
ArmR to DiaR	0.0007	0.53	0.21	1	^*^
DiaL to DiaR	0.23	0.99	0.28	1	
DiaR to ArmR	0	NaN	-0.09	0.84	^**^
DiaR to DiaL	−0.14	0.97	-0.17	1	
**MODULATORY INPUTS**					
Left Arm (patient injured Arm) to SmaR-ArmR	1.74	1	0.67	1	
Left Arm (patient injured Arm) to SmaR-DiaR	0	NaN	0.24	0.99	^**^
Right Arm to SmaL-ArmL	2.29	1	1.40	0.99	
Breathing to SmaL-DiaL	1.07	1	0.53	1	
Breathing to SmaR-DiaR	0.79	1	0.01	0.55	^*^
**DIRECT INPUT**					
Task to SmaL	0.54	1	0.82	1	
Task to SmaR	0.41	1	0.23	1	

All connections that are significant (P-value of BPA output cutoff >0.95) only for one of the groups are marked by ^*^. Connections or modulatory inputs for which the groups show a different model structure are marked by ^**^ (compare Figures 3, 4)

The following intrinsic connections were only significant for the patient group:

- *Forward connection from right arm area (ArmR) to the right diaphragm area (DiaR)*.
- *Forward connection from right supplementary motor area (SmaR) to the right arm area (DiaR)*.
- *Forward connection from left supplementary motor area (SmaL) to the left arm area (DiaL)*.
- *Forward connection from right arm area (ArmR) to the left arm area (ArmL)*.
- *Forward connection from left arm area (ArmL) to the right arm area (ArmR)*.

Figure 4 shows the intrinsic connections which were significant only in the patient group (*p* < 0.05).

The following modulation was only significant for the control group:

- *Breathing modulation to the forward connection from right supplementary motor area (SmaR) to right diaphragm area (DiaR)*.

The self-connection and the direct inputs did not show any significant behavioral differences between groups.

## Discussion

Here we provide first effective connectivity data on a rare model of peripheral reorganization—the artificial connection of an arm nerve with the phrenic nerve to allow arm movements. As previously shown, the consequence of such a procedure is the neuroplastic development of 2 homuncular representation areas for the arm in the patients' brain: the original arm area and the transformed diaphragm area. It is important to note that this kind of reorganization happens within a healthy brain and just in response to peripheral pathology. Despite direct efferences to the diseased arm are completely missing, the arm area is still highly active (large BOLD signals, Figures 1A,B), but the only way to transfer “arm movement commands” is via a connection to the diaphragm area. From a normal task fMRI data analysis however, it is not possible to tell, whether this interaction between an original and a new arm area really exists. In principle, various options for central nervous system

reorganizations are possible to allow elbow flexion after phrenic-musculocutaneous nerve connection. These include a complete independence of the old and new arm areas and even spinal reorganizations. For demonstration that the original arm area really connects to and drives the new diaphragm arm area, a DCM analysis is required. Our data indicate that this new neuroplastic cooperation between the arm and the diaphragm area indeed happens. The effective connectivity results explain how the new motor network situation works in this type of patients. The major mechanism is the generation of a driving input to the new arm output area, i.e., the right diaphragm area. This is achieved by 4 interacting mechanisms which can only be elucidated via effective connectivity analyses (compare Figures 3, 4): (1) the denervated arm area drives the diaphragm area directly, (2) the right SMA supports (1) by driving the denervated right primary arm area, (3) the left SMA drives the left primary motor cortex which supports (1) by driving the denervated right primary motor cortex and (4) the diseased (left) arm modulates direct input of the right SMA to the right diaphragm area. These 4 mechanisms form a neuroplastically transformed motor network which allows the patients to move the injured arm via activating a bifunctional diaphragm area. Note that in these patients the diaphragm area keeps its original function intact, namely breathing. This is also seen in the strong modulation of the SMA-to-diaphragm connections by breathing in patients and controls. The group difference found in the right hemisphere might be a consequence of the massive overall reorganization of the motor network in patients. In addition to the 4 major results, there was also a left-diaphragm-to-arm backward connection in the patients winning model. This fact probably reflects the establishment of a feedback loop between primary and secondary homuncular arm areas (i.e., the original and the new arm representation).

Although the DCM results well-fit to our prior task fMRI data and also functional connectivity results (in preparation), an obvious limitation of our study is the limited sample size. However, this type of surgery is rare and only a few patients are available worldwide. Consequently, patient inclusion is difficult and patients originate from various countries, partly quite distant from the investigation center. Another limitation concerns the fact, that due to their paresis, patients abilities for cooperation are limited and this resulted in some deviations from existing DCM recommendations (33). Particularly, application of a robust and blocked experimental design was required, which allowed pausing for the patients after each of the 140 s-runs [compare (36)].

In conclusion, our results provide first evidence, that peripheral nervous system reconstruction can evoke complex effective connectivity changes within the motor homunculus of healthy brains. Specifically, an essential cortex area is able to reroute its output via an auxiliary area to sustain its original motor function. This extends current knowledge about the capabilities for functional reorganization in the primary motor cortex.

## Ethics Statement

This study was approved by the ethics committee of the Medical University of Vienna and all subjects gave their written informed consents according to the Declaration of Helsinki.

## Author Contributions

AA, FF, RB, FR, and RS contributed to study conception and design. AA, FF, and EM contributed to data acquisition and analysis. AA, FF, and RB contributed to data analysis, drafting of the manuscript, and preparing the figures. AA and FF have contributed equally to this work.

### Conflict of Interest Statement

The authors declare that the research was conducted in the absence of any commercial or financial relationships that could be construed as a potential conflict of interest.
